# Cerebellum-dependent associative learning is not impaired in a mouse model of neurofibromatosis type 1

**DOI:** 10.1038/s41598-022-21429-4

**Published:** 2022-11-09

**Authors:** M. J. Ottenhoff, S. Dijkhuizen, A. C. H. Ypelaar, N. L. de Oude, S. K. E. Koekkoek, S. S.-H. Wang, C. I. De Zeeuw, Y. Elgersma, H. J. Boele

**Affiliations:** 1 Department of Neuroscience, Erasmus MC, 3000 DR Rotterdam, The Netherlands; 2 The ENCORE Expertise Center for Neurodevelopmental Disorders, Erasmus Medical Center, Rotterdam, 3015GD The Netherlands; 3 Neuroscience Institute, Princeton University, Washington Road, Princeton, NJ USA; 4 Royal Academy of Arts and Sciences (KNAW), Netherlands Institute for Neuroscience, 1105 BA Amsterdam, The Netherlands; 5 Department of Clinical Genetics, Erasmus MC, 3000 DR Rotterdam, The Netherlands

**Keywords:** Neurodevelopmental disorders, Developmental disorders, Cerebellum

## Abstract

Individuals with Neurofibromatosis type 1 (NF1) experience a high degree of motor problems. The cerebellum plays a pivotal role in motor functioning and the *NF1* gene is highly expressed in cerebellar Purkinje cells. However, it is not well understood to what extent NF1 affects cerebellar functioning and how this relates to NF1 motor functioning. Therefore, we subjected global *Nf1*^+/−^ mice to a cerebellum-dependent associative learning task, called Pavlovian eyeblink conditioning. Additionally, we assessed general motor function and muscle strength in *Nf1*^+/−^ mice. To our surprise, we found that *Nf1*^+/−^ mice showed a moderately increased learning rate of conditioned eyeblink responses, as well as improved accuracy in the adaptive timing of the eyeblink responses. Locomotion, balance, general motor function, and muscle strength were not affected in *Nf1*^+/−^ mice. Together, our results support the view that cerebellar function in *Nf1*^+/−^ mice is unimpaired.

## Introduction

Neurofibromatosis type 1 (NF1; MIM 162,200) is one of the most common monogenetic disorders (birth incidence ~ 1:2000)^1^. NF1 is caused by a heterozygous loss-of-function mutation in the *NF1* gene (MIM 613113) that encodes the protein neurofibromin. The clinical diagnosis is primarily based on distinct neurocutaneous characteristics^2,3^, yet the majority of NF1 patients also show a neurodevelopmental phenotype that affects the cognitive, behavioral and social domains^4–6^. In addition, about half of NF1 patients report problems in fine and gross motor function, as well as motor learning^7–13^. These problems in the motor domain have also been established in NF1 mouse models^14^.

The cerebellum, an essential brain structure for motor control, has been repeatedly suggested as a potential contributor to NF1-related motor problems for several reasons. First, Purkinje cells in the cerebellar cortex have high neurofibromin expression^15,16^. Second, neurofibromin is important for normal cerebellar development through its negative regulation of the RAS-ERK pathway^17,18^. Third, neurofibromin has been shown to interact with the Hyperpolarization Activated Cyclic Nucleotide Gated Potassium Channel 1 (HCN1) in an NF1 mouse model^19^. HCN1 is highly expressed in Purkinje cells and molecular layer interneurons^20,21^. Finally, NF1 patients have radiological abnormalities in the brain, the T2-weighted hyperintensities (T2Hs), for which a preferred location is the cerebellum^7,22–24^. However, these abnormalities are not present in NF1 mouse models^14^.

**Figure 1:**
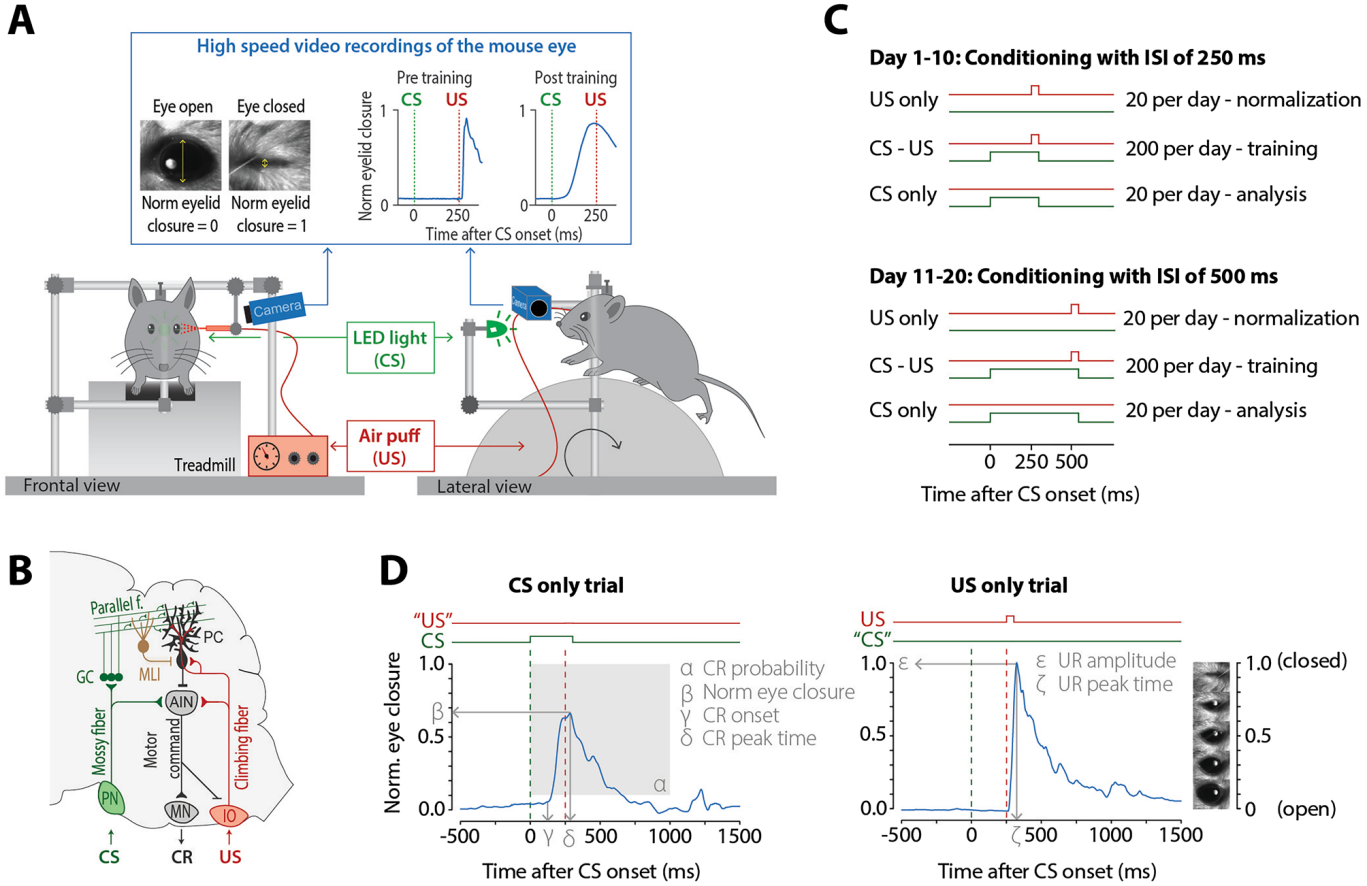
Eyeblink conditioning apparatus, training scheme, and neural circuits. (**A**) Mouse eyeblink-conditioning apparatus. Conditional stimulus (CS) is a green LED light and unconditional stimulus (US) is a mild air puff applied to the eye. Eyelid movements are measured using high-speed video recordings (350 fps). During the experiment, the animal is head-fixed, but able to move freely on a foam treadmill. (**B**) During Pavlovian EBC, memory formation takes place in PCs of defined areas of the cerebellar cortex. These PCs receive inputs from the mossy fiber–PF system, which conveys sensory CS signals (green) and input from a single CF, which transmits the instructive US signal (red). During the conditioning process, these PCs acquire a well-timed suppression of their simple spike firing in response to the CS, thereby temporarily disinhibiting the cerebellar nuclei, which drives the overt eyeblink CR. (**C**) Eyeblink conditioning training scheme. On each training day in each training phase, there are three types of trials: US only, CS–US paired, and CS only trials that are presented in a semi-randomized fashion. (**D**) Calculation of different eyeblink conditioning outcomes. *AIN* anterior interposed nucleus, *CR* conditioned response, *CS* conditional stimulus, *GC* granule cells, *IO* inferior olive, *MLI* molecular layer interneurons, *MN* motor neurons, *PC* Purkinje cell, *PN* pontine nuclei, *UR* unconditioned response, *US* unconditional stimulus.

Interestingly, NF1 patients and NF1 mouse models show normal performance on tasks that probe cerebellar function. Saccade adaptation and prism adaptation in humans^9^ as well as compensatory eye movements (VOR gain decrease and VOR phase reversal) in mice^14^ are all unaffected, suggesting that these motor problems do not have a primarily cerebellar origin. However, Pavlovian eyeblink conditioning, a sensitive and cerebellum-dependent tasks, has not been studied in the context of NF1. Eyeblink conditioning differs from VOR adaptation in that it depends on downbound rather than upbound olivocerebellar modules^25^, whereas VOR adaptation requires an increase of Purkinje cell simple-spike activity during learning, eyeblink conditioning requires mainly a decrease^26,27^. During the eyeblink conditioning task, a neutral stimulus (often a tone or light flash, called the conditional stimulus, CS) is presented a few hundred milliseconds before the onset of an air-puff to the eye (called the unconditional stimulus, US), which will elicit a blink reflex (Fig. 1). As a result of repeated CS-US pairings, subjects will eventually close their eyes in response to the CS, which is called the conditioned response (CR)^28,29^. During training both the CR proportion and the CR amplitude will gradually increase. In addition, eyeblink CRs are timed adaptively to the interval between CS and US onset, whereby the CR peaks exactly around the onset of the expected US. Thus, eyeblink conditioning measures, more than other cerebellar tasks, the millisecond-precise timing of movements.

Here, we extensively re-assessed the potential cerebellar contribution to NF1 motor problems using the cerebellar-dependent Pavlovian eyeblink conditioning task in a mouse model with a global heterozygous loss-off-function mutation in the NF1 gene (*Nf1*^+/−^ mice)^14,19,30,31^. In addition to eyeblink conditioning, we also performed tasks for general motor performance, including the ErasmusLadder, accelerating rotarod, balance beam test and grip strength test. Given the cerebellar perturbations associated with NF1 and the sensitivity of Pavlovian eyeblink conditioning for cerebellar function, we hypothesized that *global Nf1*^+/−^ mice will show alterations in eyeblink conditioning in terms of CR acquisition and performance.

## Methods and materials

### Study design and animals

For all experiments we used the heterozygote *Nf1*^+/−^ mice^14,19,30,31^. (n = 23, 12 males, 11 females) and their age- and sex-matched littermate controls (n = 24, 12 males, 12 females)*.* Each animal was considered to be an experimental unit. We calculated the sample size to be at least 17 animals per group, based on the normalized eye closure during eyeblink conditioning (primary outcome) differing 0.2 between groups, which reflects about 1 SD based on previous experiments. Additionally, for the sample size calculation the power was set at 80% and a two-sided alpha at 0.05. We increased group size from 17 animals to 23–24 animals to account for possible data loss due to surgery complications, pedestal loss or non-coping behavior.

All mice had a hybrid background of 50% C57Bl/6 and 50% 129T2/SvEms, which were previously shown to be a valid NF1 model^14,19,30–33^ and that have been used for over a decade now. These mice suffer from a heterozygote loss-of-function mutation, comparable to that found in NF1 patients. Like NF1 patients, they have deficits in synaptic plasticity leading to deficits in learning and memory formation in various neurobehavioral tests, including visual-spational motor learning. Other typical NF1 symptoms, such as peripheral neurofibromas, optic gliomas or other cutaneous conditions are less common in mice, since these symptoms are dependent from a second-hit mutation and these somatic mutations seem to occur infrequently in mice^34^. All mice were F1, aged between 15 and 25 weeks, male and female, individually housed, and received food ad libitum and subjected to 12 h:12 h light/dark cycles. Groups were age and sex matched. Apart from the age of the animals (15–25 weeks), no specific inclusion or exclusion criteria were set. All 23 *Nf1*^+/−^ mice and 24 wild-type control animals were subjected to all five types of experiments specified further down. Considering the sequence of these experiments, the eyeblink conditioning experiment was performed first, as it produces the primary outcome (normalized eye closure). Subsequently, animals were subjected to (2) the accelerated rotarod; (3) the balance beam test; (4) grip strength test; and finally (5) the ErasmusLadder. The ErasmusLadder experiment was performed last, as this is considered the most stressful experiments for the animals. All experiments were approved by the European Communities Council Directive for animal experiments and were in accordance with the Institutional (Erasmus MC) Animal Care and Use Committee guidelines. Protocols were reviewed and approved by the Erasmus Laboratory Animal Science Center (work protocol nr. 15-273-24; project license nr. AVD101002015273).

Experimenters were blind to genotype group during all experiments and primary data analysis, but not during statistical analysis. Reporting of this study is in accordance with the ARRIVE guidelines.

### Surgery

To enable head fixation during eyeblink conditioning experiments, mice underwent pedestal placement surgery. Mice were anesthetized with an isoflurane/oxygen mixture (5% for induction, 1.5–2.0% for maintenance) and body temperature was kept constant at 37 °C using a feedback measurement system (DC Temperature Control System, FHC, VS). Eyes were protected against drying using an eye lubricant (Duratears). After a local scalp injection of bupivacaine hydrochloride (2.5 mg/ml, Bupivacaine Actavis) we made a sagittal scalp incision of 2–3 cm length. Next, we carefully removed the exposed periosteum and roughened the surface of the skull using an etchant gel (Kerr, Bioggio, Switzerland). After this, we placed a small brass mounting block (1.0 × 0.4 × 0.3 mm) with 1 screw-thread and 2 additional pinholes on the skull using Optibond primer and adhesive (Kerr, Bioggio, Switzerland) and a light-curing hybrid composite Charisma (Heraeus Kulzer, Armonk, NY, USA). The surgical placement of this pedestal block allowed for head fixation during the eyeblink conditioning experiments. Surgeries typically took 15 min. After surgery, mice were given 3–5 days to recover before performing eyeblink conditioning.

### Eyeblink conditioning experimental setup

For eyeblink conditioning, mice were placed head-fixed on top of a cylindrical treadmill on which they were allowed to walk freely^27,35^. Eyelid movements were recorded using high-speed video recordings (300 fps) with a Basler camera (Basler ace 750–30 gm). All stimuli and measurement devices were controlled by National Instruments hardware and custom written LabVIEW software. The CS was a green LED of 5 mm diameter placed 10 cm in front of the mouse’s head. Because we performed our experiments in almost complete darkness, this small LED light was a salient stimulus which could be easily detected by both eyes. The US consisted of a 30 ms duration mild corneal air puff, which was controlled by an API MPPI-3 pressure injector, and delivered via a 27-gauge needle that was perpendicularly positioned at about 7 mm from the center of the left cornea (30–40 psi on pressure injector).

### Eyeblink conditioning training paradigm

*Nf1*^+/−^ mice (n = 20) and their wildtype (WT) littermates (n = 21) were trained on the Pavlovian eyeblink conditioning test. We had to exclude 6 animals in total due to inferior data quality. The reasons for this inferior quality were heterogeneous, ranging from post-surgery complications (2 sick animals) to technical issues with the eyeblink conditioning procedure (video camera or nozzle for eye puff delivery not positioned properly), leading to a missing signal (> 50% NaN) or no reflexive eyelid closures (no URs after US delivery). We would like to emphasize that our group size still exceeds the suggested group size from our sample size calculation. Mice had three days to habituate to the eyeblink setup. During the first two habituation sessions, the air puff nozzle (for US delivery) and green LED (for CS delivery) were positioned properly but no stimuli were presented. On the third day of habituation, each animal first received 20 CS-only trials and 2 US-only trials as a baseline measure, to establish that the CS did not elicit any reflexive eyelid closure. After the habituation, mice were trained for 20 consecutive daily sessions. During each session, mice received in total 240 trials separated over 20 blocks. Each block consisted of 1 CS only, 1 US only, and 10 paired CS-US trials, semi-randomly distributed over the block. A training session lasted for 45–60 min. During the first ten days of training, the duration of the CS was 280 ms and the interval between CS and US onset was 250 ms. During the second ten days of training, the duration of the CS was 530 ms and the interval between CS and US onset was 500 ms. Because of an inherent 14-ms delay in the delivery of the air puff, we triggered the air puff at 236 (for the 250 ms interstimulus interval (ISI) during day 1–10) or 484 ms (for the 500 ms ISI during day 11–20) after CS onset so that it would hit the cornea exactly at 250 ms after CS onset. The intertrial interval (ITI) was set according to the following constraints: At least 10 (± 2) seconds had to elapse, the eyelid had to be open below a predetermined threshold of 50% of a full eyelid closure, and the eyelid position had to be stable for at least 2 s for a trial to begin. During all training sessions, the experimenter carefully inspected threshold and stability parameters and adjusted them if necessary. All experiments were performed at approximately the same time of day by the same experimenter.

### Eyeblink conditioning data analysis

Individual eyeblink traces were analyzed with custom computer software (R Studio; Boston, MA, v1.3.1093). For analysis of conditioned behavior, we only included CS-only trials since they show the full kinetic profile of the eyeblink CR providing better information about the adaptive timing of eyeblink CRs. For each type of trial, a single snippet was taken from the video eyelid position signal. Each snippet, hereafter called an ‘eyeblink trace’, had a duration of 2000 ms. Eyeblink traces were filtered in forward and reverse direction with a low-pass Butterworth filter using a cut-off frequency at 50 Hz. Trials with significant activity in the 500 ms pre-CS period [> 7 times the interquartile range (IQR)] were regarded as invalid and disregarded for further analysis. Trials were min–max normalized by aligning the 500 ms pre-CS baselines and normalizing the signal so that the size of a full blink was 1 normalized eye closure (NEC). This normalization was achieved by using the reflexive blinks to the air-puff (unconditioned responses, UR) as a reference. For each session, we calculated the maximum value in the averaged UR and individual traces were normalized by dividing each trace by this value. As a consequence, in the normalized traces, an NEC of 1 corresponded with the eye being fully closed, a NEC of 0 corresponded with the eye being fully open. In valid normalized CS-only trials, A CR was defined as all eyelid movements larger than 0.1 and with a latency to CR onset of 50–500 ms and a latency to CR peak of 100–1000 ms (both relative to CS onset). Based on these, we calculated the proportion of trials that contained a CR for each session. We used the same criteria for CR detection for CS only trials given during the during day 1–10 with the 250 ms ISI and CS-only trials presented during day 11–20 with the 500 ms ISI. To analyze the effect of the shift in ISI, we calculated the amplitude (or size) of eyelid responses to the CS in two different ways: (1) The amplitude of the normalized eyelid closure at 250 ms after CS onset calculated over *all* trials (NEC_250ms_); (2) The normalized amplitude of the eyelid closure at 500 ms after CS onset calculated over *all* trials (NEC_500ms_). Finally, we looked at the adaptive timing of eyeblink CRs. For this, we determined the latency to CR onset in trials wherein a CR was present and the latency to CR peak in trials wherein a CR was present.

### ErasmusLadder

Locomotion was measured using the ErasmusLadder (Noldus, Wageningen, The Netherlands). The EramusLadder is an automated system consisting of a horizontal ladder of 37 rungs between two shelter boxes^36^. The rungs are spaced 15 mm apart and each rung is continuously monitored using an attached pressure sensor. To create a left–right alternating pattern, even rungs on one side and odd rungs on the other side are elevated by 6 mm. The shelter boxes are equipped with an LED spotlight in the roof and two pressurized air outlets in the back. During the training sessions, mice have to walk the horizontal ladder from one shelter box to the other and vice versa. The paradigm works as follows: Step 1: The mouse has to stay in the dark shelter box A for a random time interval that varies between 8 and 12 s before it is allowed to walk on the ladder rungs. Whenever the mouse wants to escape before the time interval has passed, a strong crosswind coming from the opposite shelter box B is pushing the mouse back to its starting position. Step 2a: When the time interval of 8–12 s has passed, the LED in the roof turns on and the mouse is allowed to leave the shelter box A and walk the ladder to the opposite shelter B. This LED light remains on until the mouse reaches the opposite shelter B. Step 2b: If the mouse does not leave the shelter box A within 3 s after the LED turns on, a powerful air from the back of the shelter A is activated to force the mouse to walk on the ladder. Step 3: The mouse is walking or running the ladder from box A to box B and its walking pattern is monitored. Once the mouse reaches the opposite shelter box B, the cycle starts again with step 1.

Mice were trained on 5 consecutive days, with each day consisting of 42 runs. One trial was defined as a run over the ladder from one shelter box to the other. Outcomes were the proportion of correct steps and missteps within a trial. Only proportions of correct and missteps per trial were available for analysis, not the exact count per trial. A correct step was defined as a step that is from a high rung beam to another high rung in the walking direction of the end box, irrespective of the length of this step, as mice generally prefer to walk on the upper rungs and avoid the lower ones. All other step types that involved a lower rung were considered missteps. Steps backwards were excluded from the data, since these steps were often not an actual backstep, but the tail of the mouse touching the sensors.

### Accelerating rotarod test

Mice were trained to walk on the accelerating rotarod (Ugo Basile, Comerio Varese, Italy, 7650). The rotarod was cylindrical with a diameter of 3 cm. After the mouse was placed on the rotarod, the speed gradually accelerated from 4 rotations per minute (RPM) to a maximum speed of 40 RPM over the course of 270 s. Maximum walking time was set at 300 s. Mice were trained in four consecutive days, with each day containing 4 trials. For a part of the mice the speed was increased to 80 RPM in trial 3 and 4 on day 3 and 4. The data of this 80 RPM experiment was excluded for analysis. Our outcome for analysis was the latency to fall (s) in the 40 RPM condition, which was the duration between the start of acceleration until the moment the mouse fell down from the rod or stopped walking, clinging to the rod, for three consecutive rotations.

### Balance beam test

For the balance beam test, mice had to walk over a horizontal beam of 80 cm in length that ended in their home cage. The beam had a round shape. The test was performed with two beam widths: 6 mm or 12 mm wide. Mice were trained to walk over the two beams for two consecutive days. Data of these two sessions were not recorded. During the third day, we measured the time it took the mouse to walk from start to end. Each mouse was measured twice for each beam width (4 datapoints per mouse in total). The outcome for the balance beam test is the beam crossing time in seconds.

### Grip strength test

Grip strength was measured by placing the mice with both forepaws on a grid attached to a force gauge (BIOSEB, Chaville, France), and then by steadily pulling the mice backwards by the tail. This was done twice daily for each mouse for four consecutive days. Our outcome measure was grip strength in newton, which was defined by the maximal strength produced by the mouse before releasing the grid.

### Statistical analysis

Code for statistical analysis was written in R (v4.0.3) and analysis was performed using RStudio software (v1.3.1093), supplemented with several software packages: emmeans, nlme, lme4, glmmTMB, survival, coxme and bestNormalize. All outcomes had multiple measurements per animal and timepoint or condition. Therefore, all data was analyzed using multilevel models. Depending on the type of outcome, data were analyzed with binomial logistic regression for dichotomous outcomes, Cox proportional hazards regression for survival times, multilevel linear regression models for continuous outcomes or beta regression for proportional outcomes.

Such multilevel models have several major advantages over standard parametric and non-parametric tests^37,38^. These models can accommodate the nested structure of our data (i.e., trial nested within session, session nested within animal, animal nested within group), preventing data loss by using summary measures. Additionally, in case of multilevel linear regression models, they are more robust to violations of normality assumptions and do not require homoscedasticity, which is often the case in biological data samples. Finally, multilevel models are objectively better at handling missing data points than repeated measures analysis of variance (ANOVA) models.

In our models, the main effects of categorical covariates, such as genotype, training day or their interaction, were tested with likelihood ratio tests (linear regression and beta regression) or Wald Chi-square tests (logistic regression, Cox proportional hazards regression). For each model, we tested two random effects structures: random intercepts per mouse, and random slopes for condition or time point per mouse. We used the likelihood ratio test to choose the best fitting model of the two to report. All multi-level models had an unstructured covariance matrix. Post-hoc multiple comparisons were performed for genotype pairs per condition or timepoint using the Bonferroni-Holm method. Data was considered statistically significant if p < 0.05 after correction for multiple testing. P-values are reported as exact values when > 0.0001, and are otherwise indicated as < 0.0001.

## Results

### Eyeblink conditioning

**Figure 2:**
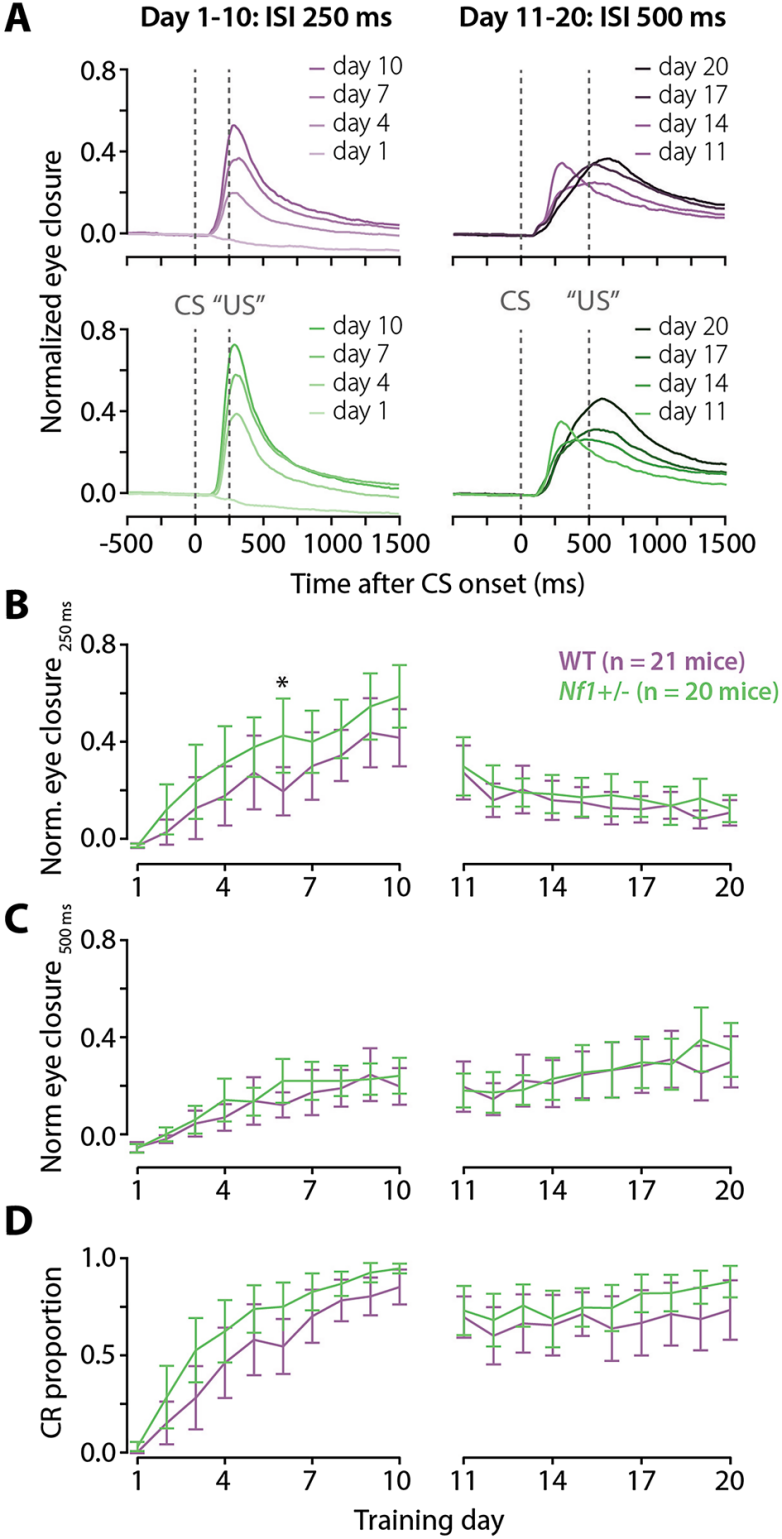
Effect of eyeblink conditioning on eyelid closure and CR proportion. (**A**) Eyeblink traces of normalized eye closure averaged per genotype and training day (first and every subsequent third day). (**B**) Normalized eye closure measured at 250 ms after the CS onset. (**C**) Averaged normalized eye closure measured at 500 ms after the CS onset. (**D**) CR proportion. The left column of figures describes the 250 ms ISI training phase, where the right column describes the subsequent 500 ms ISI training phase. Green data represents *Nf1*^+/−^ mice. Purple data represents WT mice. All error bars represent 95% confidence intervals. *p < 0.05 after correction for multiple testing.

To examine cerebellar learning, *Nf1*^+/−^ mice (n = 20) and their wildtype (WT) littermates (n = 21) were trained on the Pavlovian eyeblink conditioning test. All mice were first trained for 10 consecutive days with a relatively short interval of 250 ms between CS and US onset. Subsequently, mice were trained for another 10 days using a longer interval of 500 ms between CS and US onset (Fig. 2A). It is known that a longer interval makes the eyeblink task more difficult and harder to learn^29^. The switch from an ISI of 250 ms to 500 ms also allowed us to study the adaptation of the timing of the eyeblink CRs. We analyzed the CR proportion using a mixed effects binomial logistic regression, while normalized eye closure and timing of the CR (latency to CR onset and latency to CR peak) were analyzed using linear mixed effect models. The fixed effects were training day, genotype, and their interaction. For all mixed models, a random effects structure with training day-specific random slopes per mouse had a better fit than random intercepts per mouse only.

First, we assessed normalized eye closure at the expected time of the CR blink. The normalized eye closure at 250 ms (NEC_250ms_) in the 250 ISI training phase showed a main effect of day (LR_(9,6909)_ = 31.37, p < 0.0001), a trend for the effect of genotype (LR_(1,39)_ = 3.05, p = 0.088) and a significant effect of the day × genotype interaction (LR_(9,6809)_ = 4.93, p < 0.0001). The genotype and day × genotype effects appeared to be driven by the *Nf1*^+*/-*^ mice performing better than wild-type mice (Fig. 2B), though post-hoc pairwise comparisons revealed that this was only significant for day 6 (Table 1). The NEC_250ms_ gradually declined during the subsequent 500 ms ISI training phase (Fig. 2B). During the 500 ISI phase, the NEC_250ms_ showed a significant main effect of day (LR_(9,7113)_ = 11.26, p < 0.0001) and of the day × genotype interaction (LR_(9,7113)_ = 3.14, p = 0.00087), but not of genotype alone (LR_(1,39)_ = 0.61, p = 0.44). The significant interaction was not clearly driven by either of the genotype groups. Additionally, post-hoc pairwise comparisons between genotypes per day did not point out any significant differences between *Nf1*^+/−^ mice and WT mice (Supplementary Table 1). The normalized eye closure at 500 ms (NEC_500ms_) after the CS onset gradually increased over the 500 ISI training phase (Fig. 2C). The NEC_500ms_ showed a significant main effect of day (LR_(9,7113)_ = 4.63, p < 0.0001) and of the day × genotype interaction (LR_(9,7113)_ = 5.42, p < 0.001), but not of genotype (LR_(1,39)_ = 0.116, p = 0.74). Again, the significant interaction effect was not clearly driven by one group, with post-hoc testing revealing no significant differences between genotypes per day (Supplementary Table 2).

**Table 1:** Normalized eye closure at 250 ms after CS onset for the 250 ISI training phase.

Day	WT (n = 21)	*Nf1*^+/−^ (n = 20)	Comparisons^†^		
	NEC_250ms_, mean (SD)	NEC_250ms_, mean (SD)	Estimated difference [95% CI]	p-value^†^	Adjusted p-value
1	− 0.02 (0.02)	− 0.02 (0.01)	0.00 [− 0.09, 0.09]	0.95	1.00
2	0.02 (0.09)	0.10 (0.18)	0.07 [− 0.02, 0.17]	0.12	1.00
3	0.1 (0.22)	0.19 (0.26)	0.08 [− 0.01, 0.18]	0.08	0.81
4	0.14 (0.21)	0.25 (0.25)	0.10 [0.00, 0.20]	0.05	0.51
5	0.22 (0.27)	0.30 (0.21)	0.08 [− 0.03, 0.18]	0.15	1.00
6	0.16 (0.17)	0.34 (0.26)	0.18 [0.06, 0.29]	0.003	0.03*
7	0.24 (0.24)	0.32 (0.22)	0.08 [− 0.04, 0.20]	0.20	1.00
8	0.28 (0.19)	0.36 (0.21)	0.09 [− 0.04, 0.22]	0.17	1.00
9	0.35 (0.25)	0.44 (0.23)	0.09 [− 0.05, 0.23]	0.21	1.00
10	0.33 (0.21)	0.47 (0.22)	0.14 [− 0.02, 0.29]	0.08	0.80

*Significant difference between genotypes of p < 0.05. ^†^The degrees of freedom for all comparisons is 39. *WT* wild type*.*

Second, we assessed the CR proportion that showed a significant effect of day (X^2^_(9)_ = 32.34, p < 0.0001), genotype (X^2^_(1)_ = 5.26), p = 0.022) and the day × genotype interaction (X^2^_(9)_ = 2.23, p = 0.017). While the significant genotype and interaction effects seemed to be driven by a steeper increase in CR proportion in the *Nf1*^+/−^ mice with training (Fig. 2D), none of the post-hoc genotype comparisons were significant (Table 2). During the subsequent 500 ms ISI training phase, the CR proportion showed a significant effect of day (X^2^_(9)_ = 6.24, p < 0.0001), but not of genotype (X^2^_(1)_ = 2.31, p = 0.13) or the day × genotype interaction (X^2^_(9)_ = 1.24, p = 0.26) (Fig. 2D, Supplementary Table 3). In summary, *Nf1*^+/−^ mice appeared to learn the eyeblink conditioning task slightly quicker than wild-type control animals.

**Table 2:** CR proportion in the 250 ISI training phase.

Day	WT (n = 21)	*Nf1*^+*/-*^ (n = 20)	Comparisons		
	CR proportion, median (q25–q75)	CR proportion, median (q25–q75)	*Nf1*^+/−^/WT OR [95% CI]	p-value	Adjusted p-value
1	0.00 (0.00–0.00)	0.00 (0.00–0.06)	35.3 [1.82, 683.88]	0.02	0.18
2	0.00 (0.00–0.20)	0.04 (0.00–0.63)	5.23 [0.82, 33.24]	0.08	0.80
3	0.00 (0.00–0.62)	0.55 (0.23–0.89)	6.31 [1.24, 32.19]	0.03	0.27
4	0.39 (0.00–0.89)	0.79 (0.36–0.91)	2.91 [0.69, 12.35]	0.15	1.00
5	0.63 (0.00–0.89)	0.79 (0.73–0.88)	2.36 [0.65, 8.52]	0.19	1.00
6	0.50 (0.39–0.82)	0.80 (0.69–0.90)	3.27 [1.05, 10.20]	0.04	0.41
7	0.78 (0.47–0.95)	0.88 (0.69–0.94)	1.69 [0.60, 4.75]	0.32	1.00
8	0.85 (0.57–0.94)	0.90 (0.84–0.95)	2.16 [0.81, 5.77]	0.13	1.00
9	0.88 (0.63–1.00)	0.95 (0.89–1.00)	2.51 [0.94, 6.75]	0.07	0.67
10	0.88 (0.85–1.00)	0.95 (0.90–1.00)	3.69 [1.25, 10.83]	0.02	0.18

An odds ratio (OR) > 1 indicates a higher CR proportion in the *Nf1*^+/−^ mice over the wild type (WT) mice.

**Figure 3:**
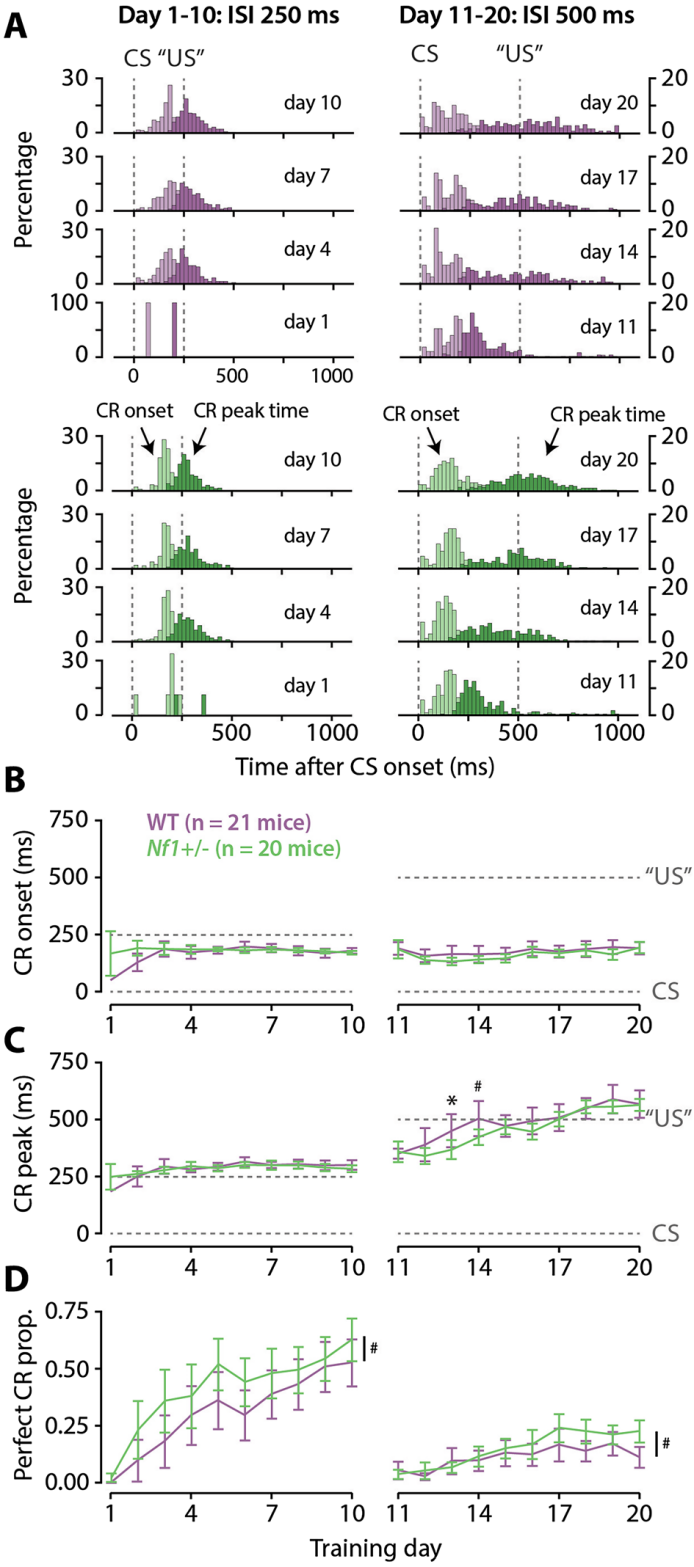
Timing of conditioned eyeblink responses. (**A**) Histograms of the timing of conditioned responses (CR) per genotype and training day (first and subsequent third day). (**B**) Average timing of CR onset. (**C**) timing of CR peak. The left column of figures describes the 250 ms ISI training phase, where the right column describes the subsequent 500 ms ISI training phase. (**D**) Proportion of perfectly timed CRs, i.e., CRs whereby the peak fell within a window of max 50 ms plus or minus the omitted US onset. Green data represents *Nf1*^+/−^ mice. Purple data represents WT mice. ^#^Trend towards a significant difference between genotypes of p < 0.1; *Significant difference between genotypes of p < 0.05. All error bars represent 95% confidence intervals.

Third, we looked in detail at the adaptive timing of eyeblink CRs (Fig. 3A). For this analysis, we only included trials wherein a CR was present, following the same criteria we used for calculating the CR proportion per session. The latency to CR onset showed a significant effect of day during the 250 ms ISI training phase (LR_(9,3891)_ = 6.45, p < 0.0001) and of the day × genotype interaction (LR_(9,3891)_ = 3.85, p < 0.0001), but not of genotype (LR_(1,39)_ = 2.62, p = 0.11). The significant interaction effect was not clearly driven by one of the groups (Fig. 3B), and the post-hoc comparisons did not show any significant pairwise comparisons between genotypes per day (Supplementary Table 4). During the 500 ms ISI training phase, the latency to CR onset showed a significant effect of day (LR_(9,4915)_ = 12.80, p < 0.0001), but not of genotype (LR_(1,39)_ = 2.05, p = 0.16) or the day × genotype interaction (LR_(9,4915)_ = 1.21, p = 0.28) (Fig. 3B, Supplementary Table 5). The latency to CR peak showed a significant effect of training day during the initial 250 ms training phase (LR_(9,3886)_ = 6.38, p < 0.0001), but not of genotype (LR_(1,39)_ = 2.45, p = 0.13) or the day × genotype interaction (LR_(9,2886)_ = 1.29, p = 0.24) (Fig. 3C, Supplementary Table 6). During the subsequent 500 ms ISI training phase, the latency to CR peak showed a significant effect of day (LR_(9,4911)_ = 27.20, p < 0.0001) and of the day × genotype interaction (LR_(9,4911)_ = 3.74, p = 0.00011), but not of genotype (LR_(1,39)_ = 1.76, p = 0.19). The significant interaction seemed to be caused by a more rapid initial increase in the WT mice (Fig. 3C), which was confirmed by the post-hoc comparisons showing a significant longer CR peak latency for the WT mice on day 3 and a trend for day 4 (Table 3).

**Table 3:** Latency to CR peak in the 500 ISI training phase.

Day	WT		*Nf1*^+/−^		Comparisons^†^		
	N	CR peak time (s), mean (SD)	N	CR peak time (s), mean (SD)	Estimated difference (s) [95% CI]	p-value	Adjusted p-value
11	21	350 (48)	20	358 (97)	4 [− 49, 56]	0.89	1.00
12	20	389 (156)	20	340 (75)	− 17 [− 68, 33]	0.49	1.00
13	20	450 (155)	20	367 (89)	− 73 [− 121, − 26]	0.003	0.03*
14	20	504 (165)	19	422 (71)	− 65 [− 112, − 19]	0.007	0.07^#^
15	21	571 (104)	20	467 (70)	3 [− 42, 48]	0.88	1.00
16	18	493 (119)	20	446 (76)	− 54 [− 100, − 8]	0.02	0.23
17	17	508 (115)	20	501 (68)	− 8 [− 55, 38]	0.72	1.00
18	19	548 (94)	20	555 (61)	9 [− 39, 57]	0.71	1.00
19	19	589 (131)	20	556 (60)	− 14 [− 64, 36]	0.58	1.00
20	19	568 (126)	20	564 (57)	− 14 [− 67, 39]	0.60	1.00

Latency relative to CS onset, which is set at 0 ms. ^†^The degrees of freedom for all comparisons is 39. ^#^Trend towards a significant difference between genotypes of p < 0.1.*Significant difference between genotypes of p < 0.05. *WT* wild type.

Last, we examined the proportion of “perfectly timed” CRs, defined as occurring within a symmetric 100 ms window around the timing of the omitted US (day 1–10, 200–300 ms after CS; day 11–20, 450–550 ms after CS). During the 250 ms ISI training phase, the proportion of these perfectly timed CRs showed a significant effect for day (X^2^_(9)_ = 14.76, p =  < 0.0001) and for day × genotype interaction (X^2^_(9)_ = 2.04, p = 0.032), though not for genotype (X^2^_(1)_ = 0,001, p = 0.97). While the *Nf1*^+/−^ mice appear to show a steeper increase in CR proportion in the beginning of the training phase (Fig. 3D), none of the post-hoc genotype comparisons per training day were significant (Supplementary Table 7). For the 500 ms ISI training phase, the proportion of perfectly timed CRs showed a significant effect for both genotype (X^2^_(1)_ = 3.94, p = 0.047), day (X^2^_(9)_ = 13.83, p < 0.0001), and day × genotype (X^2^_(9)_ = 2.08, p = 0.027). The *Nf1*^+*/-*^ mice appear to show a higher proportion of CRs towards the end of the training, which is supported by the post-hoc comparisons showing a trend for day 18 and a significant effect for day 20 (Table 4).

**Table 4:** “Perfectly timed” CR proportion in the 500 ms ISI training phase.

Day	WT (n = 21)	*Nf1*^+/−^ (n = 20)	Comparisons		
	CR (%), median (q25–q75)	CR (%), median (q25–q75)	*Nf1*^+/−^/WT OR [95%CI]	p-value	Adjusted p-value
11	0.06 (0.00–0.06)	0.00 (0.00–0.06)	0.80 [0.33, 1.95]	0.63	1.00
12	0.00 (0.00–0.06)	0.00 (0.00–0.06)	2.49 [1.00, 6.23]	0.05	0.35
13	0.00 (0.00–0.15)	0.06 (0.05–0.07)	0.80 [0.39, 1.62]	0.53	1.00
14	0.06 (0.00–0.20)	0.10 (0.06–0.17)	1.50 [0.78, 2.89]	0.22	0.90
15	0.11 (0.06–0.16)	0.13 (0.09–0.21)	1.43 [0.78, 2.64]	0.25	0.90
16	0.12 (0.00–0.20)	0.14 (0.06–0.27)	1.77 [0.96, 3.26]	0.07	0.39
17	0.13 (0.00–0.29)	0.25 (0.11–0.32)	1.91 [1.07, 3.39]	0.03	0.22
18	0.17 (0.06–0.22)	0.19 (0.16–0.28)	2.21 [1.23, 3.99]	0.01	0.07^#^
19	0.21 (0.07–0.26)	0.22 (0.15–0.26)	1.51 [0.84, 2.72]	0.16	0.82
20	0.11 (0.00–0.21)	0.22 (0.13–0.27)	2.92 [1.57, 5.42]	0.00	0.01*

An odds ratio (OR) > 1 indicates a higher CR proportion in the *Nf1*^+/−^ mice over the wild type (WT) mice. ^#^Trend towards a significant difference between genotypes of p < 0.1. *Significant difference between genotypes of p < 0.05.

In summary, these results indicate that *Nf1*^+/−^ mice have a modestly increased performance in eyeblink conditioning compared with wild-type controls. *Nf1*^+/−^ mice showed a slightly larger eyelid closure during the 250 ms ISI training phase and a higher CR proportion in the 250 ms ISI training phase. In addition, we found that the adaptive timing of eyeblink CRs was slightly improved in *Nf1*^+/−^ mice. Additionally, the proportion of “perfectly timed” CRs in the 500 ms ISI training phase was increased in *Nf1*^+/−^ mice towards the end of the training. Thus, cerebellar associative learning capacity was modestly enhanced in *Nf1*^+/−^ mice.

### ErasmusLadder

**Figure 4:**
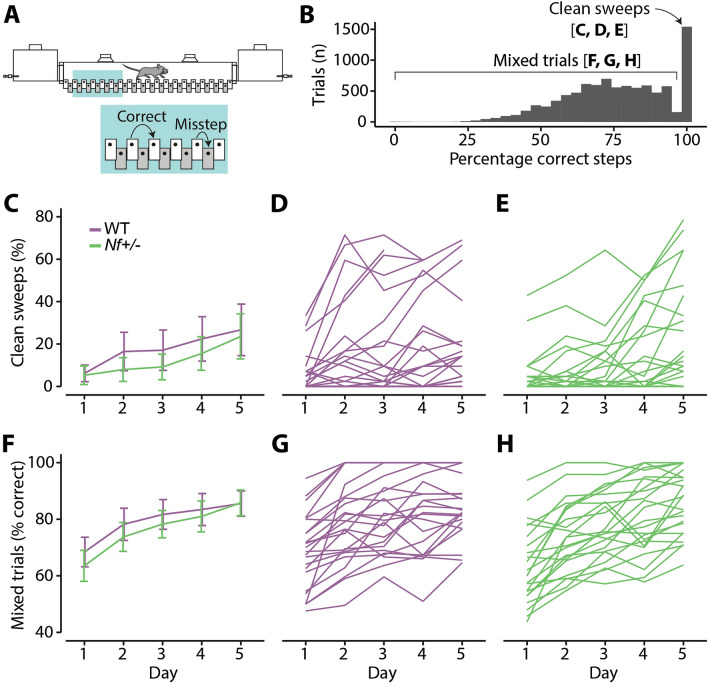
ErasmusLadder performance. (**A**) Schematic of the ErasmusLadder task. Correct steps are defined as a step from a high beam to any high beam further towards the end of the ladder. Missteps are defined as any step involving a low beam. (**B**) Histogram of the percentage of correct steps per trial. There is a large number of trials that have 100% correct steps, the so-called clean sweeps. The other trials are called mixed trials. (**C**) Lines represent the mean of the individual median proportion of clean sweeps, with whiskers being the 95% CI. In (**D**) and (**E**) each line represents the median proportion of clean sweeps of an individual mouse. (**F**) Lines represent the means of the individual median proportion of correct steps within mixed trials, with whiskers being the 95% CI. In (**G**) and (**H**) each line represents the median proportion of correct steps within mixed trials of an individual mouse.

The ErasmusLadder assesses locomotion patterns in mice (Fig. 4A). *Nf1*^+/−^ mice (n = 23) and their wildtype littermates (n = 24) were trained on the ErasmusLadder for 5 consecutive days. Each training day consisted of 42 trials, whereby one trial was defined as a run over the ladder from one shelter box to the other. For each trial, we analyzed the proportion of correct steps versus missteps, as there was no original count data available. Upon data inspection, we observed that the proportion of correct steps outcome had a two-part distribution: a considerable portion of trials that contained correct steps only (16% of trials), while the other part of the data had a mixture of correct steps and missteps, with no clear continuum between those parts (Fig. 4B). We refer to correct steps only trials as ‘clean sweeps’. This two-part distribution required a two-model approach.

First, we analyzed the proportion of correct steps in all trials in a dichotomous fashion (clean sweep or not) with a multi-level binomial logistic regression. The fixed effects in this model were training day, genotype and their interaction. The random structure were day-specific random slopes per mouse. We observed a significant main effect of day (X^2^_(4)_ = 14.40, p < 0.0001) and of the interaction between day and genotype (X^2^_(4)_ = 2.58, p = 0.035), though not of genotype alone (X^2^_(1)_ = 0.49, p = 0.48). Post-hoc comparisons indicated the largest differences between genotypes were impairments in *Nf1*^+*/-*^ mice on day 2, 3 and 4, though none of the comparisons were significantly different (Fig. 4C–E, Supplementary Table 8).

Second, we analyzed the trials containing both correct steps and missteps separately, thus excluding clean sweep trials. This analysis concerned 7738 out of 9228 trials. Since we were dealing with a proportion measure for this analysis, we used a multi-level beta regression. The fixed effects in this model were day, genotype and their interaction, with the random structure being random slopes for day per mouse. This model had a significant main effect of day (LR_(4,7724)_ = 66.92, p < 0.0001), while neither genotype (LR_(1,7724)_ = 0.80, p = 0.37) nor the interaction between day and genotype (LR_(4,7724)_ = 0.27, p = 0.90) were significant (Fig. 4F–H, Supplementary Table 9). Together, these results indicate that the *Nf1*^+*/-*^ showed equally good locomotion and gross motor function on the ErasmusLadder compared to their wildtype littermates.

### Accelerating rotarod

**Figure 5:**
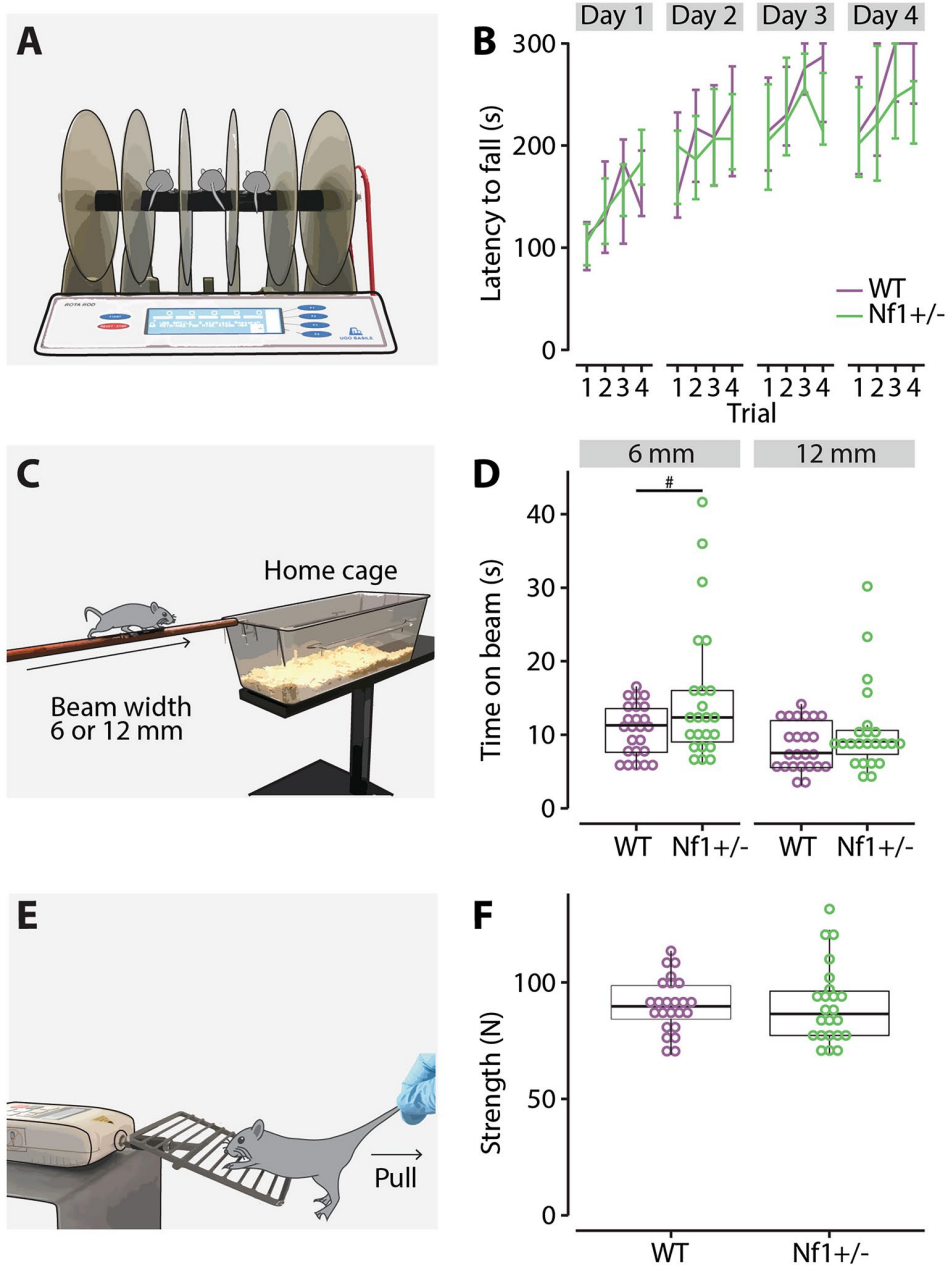
Performance on the accelerated rotarod, balance beam and grip test. (**A**) Illustration of the accelerating rotarod test, where mice are placed on an accelerating rotating rod. The latency to fall on the lever below the rod is measured up to 300 s. (**B**) Lines represent the median latency to fall (s) per genotype, with the whiskers displaying the interquartile range. (**C**) Illustration of the balance beam test, where it was timed how long mice took to walk from start to end on two beam widths (6 mm and 12 mm). (**D**) Box plots of the median time on beam per beam width and genotype (s). Each dot represents the median of a single mouse over two trials. (**E**) Illustration of the grip test, where the max strength (N) is measured a mouse uses to grab onto a grid before it lets go. (**F**) Boxplots of the median strength per genotype. Each dot represents the median of an individual mouse over eight trials.

To further examine gross motor learning, *Nf1*^+/−^ mice (n = 23) and their WT littermates (n = 24) were trained on the accelerating rotarod test (Fig. 5A). We only analyzed data for the 40 rpm experiments, leaving only 9 *Nf1*^+/−^ mice and 9 wildtype mice for trial 3 and 4 of day 3 and 4 (see “Methods”, Supplementary Table 10). We analyzed the latency to fall (s) with a multilevel Cox proportional hazards model, as this outcome is censored at 300 s and is essentially a survival time till fall event. The fixed effects for this model were the training day, genotype, and their interaction, with the random effects being random slopes per mouse. Both genotype groups show an increase of the latency to fall with training day (Fig. 5B). The main effect of day was significant (X^2^_(3)_ = 587.55, p < 0.0001), but there was no significant main effect of genotype (X^2^_(1)_ = 0.006, p = 0.94) or the interaction between day and genotype (X^2^_(3)_ = 4.40, p = 0.22) (Supplementary Table 11). In summary, both the *Nf1*^+/−^ mice and their WT littermates performed equally well on the accelerating rotarod test.

### Balance beam

To further test gross motor function, *Nf1*^+/−^ mice (n = 23) and their WT littermates (n = 24) performed the balance beam test (Fig. 5C). We analyzed the time it took for the mice to walk from start to the end on two different beams with widths of 6 mm and 12 mm using a multilevel linear regression model. As the model residuals appeared to have a positive skew, we performed a log-transformation on the outcome. The fixed effects for the model were beam width, genotype and their interaction, with the random effects being random intercepts per mouse. There was a significant main effect of beam width (F_(1,139)_ = 66.27, p < 0.0001) and a trend for genotype (F_(1,45)_ = 3.41, p = 0.071), while their interaction was not significant (F_(1,139)_ = 0.83, p = 0.36) (Fig. 5D). Post-hoc paired comparisons revealed a trend for the difference between genotypes within the 6 mm beam condition, while there was no difference for the 12 mm condition (Supplementary Table 12). These results, therefore, suggest that *Nf1*^+/−^ and WT mice show comparable gross motor function on the balance beam test.

### Grip strength

Motor function can be greatly impacted by muscle strength. We therefore tested the *Nf1*^+/−^ mice (n = 23) and their WT littermates (n = 24) on the grip strength test, which tests the peak strength in the front paws of the animals (Fig. 5E). Peak strength (N) was analyzed using a multilevel linear regression model. We observed that the residuals of this model showed a prominent positive skew, that was resolved by a log-transformation of the outcome. From the linear regression model, we estimate the back-translated peak strength in the *Nf1*^+/−^ mice to be 89.64 N (95% CI [84.09, 95.55]) and in the WT mice to be 91.42 N (95% CI [85.87, 97.32]), which did not significantly differ (estimated difference = − 1.77 N, 95% CI [− 9.87, 6.32]; t(45) = 0.66, p = 0.66) (Fig. 5F). These results indicate that the *Nf1*^+/−^ mice had comparable peak strength to WT mice, suggesting that any change in motor function is unlikely to be a result of altered muscle peak strength.

## Discussion

We found that cerebellar associative learning, tested with Pavlovian eyeblink conditioning, tends to be improved in mice with a global heterozygous loss-of-function mutation in the NF1 gene (Figs. 2, 3). These *Nf1*^+/−^ mice showed no decrease in CR proportion nor CR amplitude in the initial 250 ms ISI training phase (Fig. 2, left panels); the minor significant effect we found was actually in favor of the *Nf1*^+/−^ mice. When the ISI was shifted from 250 ms to a more difficult one of 500 ms, WT mice adapted the timing of their CRs in fewer trials to the new ISI (Fig. 3C). However, this may be caused by the *Nf1*^+/−^ mice showing larger eyeblink CR amplitudes at the end of the 250 ms training phase and that extinction of these larger CRs simply took more time (Fig. 2B). Adaptation of eyeblink CRs to a new ISI is essentially an extinction of the old CRs together with acquisition of the CR for the new ISI^29,39^. Interestingly, the proportion of “perfectly timed” CRs, which we defined as CRs occurring within a symmetric 100 ms window around the onset of the omitted US, was also significantly higher in *Nf1*^+/−^ mice during the 500 ms ISI (Fig. 3D). This finding indicates that *Nf1*^+/−^ mice were better at timing their conditioned blinks, providing better protection of their eyes to the aversive US. Taken together, our eyeblink conditioning experiments indicate that cerebellar associative learning in *Nf1*^+/−^ mice is intact or improved.

In addition to eyeblink conditioning, we assayed tasks that screen for general motor performance, including the accelerating rotarod (Fig. 5A,B), balance beam (Fig. 5C,D), and grip strength (Fig. 5E,F). *Nf1*^+/−^ mice did not show any motor deficits in any of these tests. Although the effects on most of these tests predominantly pointed towards a better performance in the WT mice over the *Nf1*^+/−^ mice, they did not reach significance despite a relatively large group size. In contrast to our findings, the accelerated rotarod and grip test applied to *Nf1*^+/−^ mice in earlier studies using slightly smaller group sizes did show significant deficits^14,19^. However, both these studies exhibited effect sizes that point towards relatively small deficits. Our current findings on a cerebellum-dependent locomotion task, the ErasmusLadder task, also did not show any significant deficit (Fig. 4). Previous studies using other cerebellum-specific behavioral tasks, such as compensatory eye movement tasks in mice^14^ or saccade and prism adaptation tasks in human^9^, also could not detect any performance or learning deficits. As such, our findings are in line with most previous NF1 studies, adding to the possibility that the motor problems associated with NF1 are relatively minor^4,12^ and may originate at least partly from an extracerebellar source.

The combination of these findings raises the question of why the eyeblink conditioning parameters came to be improved in *Nf1*^+/−^ mice. GABA-mediated inhibition is enhanced in *Nf1*^+/−^ mice^31^, and since eyeblink conditioning is predominantly mediated by suppression of simple spike activity of Purkinje cells in the downbound microzones^26,40^, and GABAergic inhibition by molecular layer interneurons contributes to this suppression^27^, it is attractive to speculate that eyeblink conditioning is improved because of an enhanced impact of GABA. This effect could be further enhanced by the fact that HCN1 channels, which are activated by GABA-mediated hyperpolarization, are attenuated by the NF1 mutation^19,41^. Enhanced GABA-mediated inhibition in NF1 may also explain why some of the minor motor symptoms might be extracerebellar, as enhanced GABAergic activity in areas like the cerebral cortex may induce behavioral phenotypes^31^. In addition, extracerebellar structures such as amygdala, hippocampus, and prefrontal cortex play important (modulatory) roles in eyeblink conditioning^42–46^.

In conclusion, we find that *Nf1*^+/−^ mice show moderately increased learning rate of conditioned eyeblink responses, and the adaptive timing of those responses was also slightly more accurate. Locomotion, balance, general motor function, and muscle strength were not affected. Future studies will have to elucidate whether these observations can be explained by a differential impact of NF1 on GABA-mediated processes in downbound and upbound microzones in the cerebellar cortex.

## Supplementary Information

Supplementary Tables.

Supplementary Information 2.

Supplementary Information 3.

Supplementary Information 4.

Supplementary Information 5.

Supplementary Information 6.

## Data Availability

The datasets used in this study are included as supplementary material: Supplementary Material 13 (eyeblink conditioning data); Supplementary Material 14 (ErasmusLadder data); Supplementary Material 15 (accelerating rotarod data); Supplementary Material 16 (balance beam data); Supplementary Material 17 (grip test data).
