# Supplementary material for: Cerebellum-dependent associative learning is not impaired in a mouse model of neurofibromatosis type 1
Source: Sci Rep. 2022 Nov 9;12:19041. doi: 10.1038/s41598-022-21429-4 (PMC9646701; doi:10.1038/s41598-022-21429-4)
Supplement: Supplementary file 1 — Supplementary Tables. [file 41598_2022_21429_MOESM1_ESM.docx]

### Supplementary Tables

#### Supplementary Table 1. Normalized eye closure at 250 ms after CS onset for the 500 ISI training phase.

|  | WT (n=21) | *Nf1^+/-^* (n=20) | Comparisons^†^ | | |
| --- | --- | --- | --- | --- | --- |
| Day | NEC_250ms_,  mean (SD) | NEC_250ms_,  mean (SD) | Estimated difference  [95%CI] | P-value | Adjusted  p-value |
| 11 | 0.22 (0.19) | 0.24 (0.20) | 0.01 [-0.08, 0.10] | 0.77 | 1.00 |
| 12 | 0.13 (0.12) | 0.17 (0.14) | 0.05 [-0.04, 0.13] | 0.27 | 1.00 |
| 13 | 0.16 (0.17) | 0.15 (0.10) | -0.01 [-0.09, 0.07] | 0.80 | 1.00 |
| 14 | 0.13 (0.14) | 0.15 (0.13) | 0.02 [-0.05, 0.09] | 0.57 | 1.00 |
| 15 | 0.12 (0.11) | 0.14 (0.13) | 0.02 [-0.05, 0.08] | 0.65 | 1.00 |
| 16 | 0.10 (0.12) | 0.14 (0.15) | 0.04 [-0.03, 0.10] | 0.24 | 1.00 |
| 17 | 0.10 (0.09) | 0.13 (0.12) | 0.03 [-0.03, 0.09] | 0.28 | 1.00 |
| 18 | 0.11 (0.10) | 0.11 (0.13) | 0.00 [-0.06, 0.05] | 0.93 | 1.00 |
| 19 | 0.06 (0.06) | 0.13 (0.14) | 0.07 [0.01, 0.12] | 0.019 | 0.19 |
| 20 | 0.09 (0.09) | 0.10 (0.09) | 0.01 [-0.04, 0.07] | 0.62 | 1.00 |

*† The degrees of freedom for the comparisons is 39. WT = wild type.*

#### Supplementary Table 2. Normalized eye closure at 500 ms after CS onset for the 500 ISI training phase.

|  | WT | | *Nf1^+/-^* | | Comparisons^†^ | | |
| --- | --- | --- | --- | --- | --- | --- | --- |
| Day | N | NEC_500ms_,  mean (SD) | N | NEC_500ms_,  mean (SD) | Estimated difference  [95%CI] | P-value | Adjusted  p-value |
| 11 | 21 | -0.04 (0.04) | 20 | -0.05 (0.03) | -0.02 [-0.1, 0.07] | 0.68 | 1.00 |
| 12 | 21 | -0.01 (0.03) | 20 | 0.00 (0.05) | 0.02 [-0.06, 0.11] | 0.55 | 1.00 |
| 13 | 21 | 0.04 (0.10) | 20 | 0.05 (0.10) | -0.03 [-0.11, 0.05] | 0.48 | 1.00 |
| 14 | 21 | 0.06 (0.10) | 19 | 0.12 (0.15) | 0.02 [-0.07, 0.10] | 0.69 | 1.00 |
| 15 | 21 | 0.11 (0.18) | 20 | 0.11 (0.10) | 0.01 [-0.08, 0.10] | 0.88 | 1.00 |
| 16 | 21 | 0.10 (0.09) | 20 | 0.18 (0.16) | 0.00 [-0.10, 0.09] | 0.93 | 1.00 |
| 17 | 21 | 0.14 (0.17) | 20 | 0.18 (0.14) | 0.01 [-0.09, 0.12] | 0.81 | 1.00 |
| 18 | 21 | 0.16 (0.14) | 20 | 0.18 (0.11) | -0.01 [-0.12, 0.10] | 0.81 | 1.00 |
| 19 | 21 | 0.20 (0.20) | 20 | 0.19 (0.11) | 0.11 [-0.01, 0.23] | 0.065 | 0.65 |
| 20 | 21 | 0.16 (0.14) | 20 | 0.20 (0.13) | 0.04 [-0.09, 0.17] | 0.51 | 1.00 |

*† The degrees of freedom for the comparisons is 39. WT = wild type.*

#### Supplementary Table 3. CR proportion in the 500 ISI training phase.

|  | WT (n=21) | *Nf1^+/-^* (n=20) | Comparisons | | |
| --- | --- | --- | --- | --- | --- |
| Day | CR proportion,  median (q25-q75) | CR proportion,  median (q25-q75) | *Nf1^+/-^* / WT  OR [95%CI] | P-value | Adjusted  p-value |
| 1 | 72 (50-87) | 80 (49-94) | 1.39 [0.55, 3.53] | 0.49 | 1.00 |
| 2 | 67 (29-82) | 68 (47-89) | 1.83 [0.71, 4.74] | 0.21 | 1.00 |
| 3 | 62 (43-94) | 71 (55-89) | 2.12 [0.79, 5.72] | 0.14 | 1.00 |
| 4 | 63 (44-89) | 72 (35-100) | 1.56 [0.55, 4.46] | 0.40 | 1.00 |
| 5 | 72 (50-94) | 67 (49-94) | 1.49 [0.49, 4.57] | 0.48 | 1.00 |
| 6 | 76 (27-88) | 77 (52-96) | 2.62 [0.79, 8.66] | 0.11 | 1.00 |
| 7 | 78 (56-94) | 80 (65-96) | 3.01 [0.83, 10.91] | 0.09 | 0.93 |
| 8 | 83 (56-100) | 86 (61-100) | 2.87 [0.72, 11.48] | 0.14 | 1.00 |
| 9 | 82 (44-94) | 92 (59-100) | 3.8 [0.86, 16.82] | 0.08 | 0.78 |
| 10 | 88 (68-94) | 92 (78-100) | 4.68 [0.94, 23.23] | 0.06 | 0.59 |

*OR = odds ratio; WT = wild type.*

#### Supplementary Table 4. Latency to CR onset for the 250 ISI training phase*.*

|  | WT | | *Nf1^+/-^* | | Comparison^†^ | | |
| --- | --- | --- | --- | --- | --- | --- | --- |
| Day | N | CR onset (s), mean  (SD) | N | CR onset (s),  mean (SD) | Estimated difference (s)  [95%CI] | P-value | Adjusted  p-value |
| 1 | 1 | 50 (NA) | 5 | 167 (79) | 134 [20, 249] | 0.02 | 0.23 |
| 2 | 9 | 128 (50) | 10 | 191 (46) | 30 [2, 59] | 0.04 | 0.38 |
| 3 | 10 | 187 (47) | 17 | 187 (51) | 1 [-22, 24] | 0.93 | 1.00 |
| 4 | 14 | 172 (49) | 17 | 185 (38) | 6 [-15, 27] | 0.56 | 1.00 |
| 5 | 15 | 182 (27) | 18 | 185 (17) | 3 [-15, 22] | 0.73 | 1.00 |
| 6 | 19 | 198 (44) | 19 | 181 (25) | -17 [-35, 0] | 0.06 | 0.56 |
| 7 | 20 | 191 (39) | 20 | 184 (23) | -6 [-23, 11] | 0.46 | 1.00 |
| 8 | 21 | 179 (44) | 20 | 181 (20) | 1 [-16, 17] | 0.92 | 1.00 |
| 9 | 21 | 168 (44) | 20 | 177 (19) | 7 [-10, 24] | 0.41 | 1.00 |
| 10 | 21 | 179 (27) | 20 | 170 (17) | -5 [-23, 12] | 0.54 | 1.00 |

*† The degrees of freedom for all comparisons is 39. WT = wild type.*

#### Supplementary Table 5. Latency to CR onset in the 500 ISI training condition.

|  | WT | | *Nf1^+/-^* | | Comparisons | | |
| --- | --- | --- | --- | --- | --- | --- | --- |
| Day | N | CR onset (s),  mean (SD) | N | CR onset (s),  mean (SD) | Estimated difference (s)  [95%CI] | P-value* | Adjusted  p-value |
| 1 | 21 | 189 (59) | 20 | 186 (84) | -16 [-47, 16] | 0.32 | 1.00 |
| 2 | 20 | 158 (58) | 20 | 140 (33) | -16 [-47, 15] | 0.30 | 1.00 |
| 3 | 20 | 165 (75) | 20 | 134 (34) | -23 [-52, 7] | 0.13 | 1.00 |
| 4 | 20 | 165 (75) | 19 | 142 (32) | -18 [-47, 10] | 0.20 | 1.00 |
| 5 | 21 | 167 (55) | 20 | 147 (38) | -23 [-51, 5] | 0.10 | 1.00 |
| 6 | 18 | 188 (63) | 20 | 174 (48) | -23 [-51, 5] | 0.11 | 1.00 |
| 7 | 17 | 176 (50) | 20 | 169 (34) | -8 [-36, 20] | 0.56 | 1.00 |
| 8 | 19 | 187 (70) | 20 | 181 (53) | -9 [-38, 19] | 0.52 | 1.00 |
| 9 | 19 | 195 (62) | 20 | 163 (48) | -30 [-59, 0] | 0.05 | 0.48 |
| 10 | 19 | 191 (55) | 20 | 193 (51) | 0 [-30, 31] | 0.98 | 1.00 |

*WT = wild type.*

#### Supplementary Table 6. Latency to CR peak for the 250 ISI training condition.

|  | WT | | *Nf1^+/-^* | | Comparisons^†^ | | |
| --- | --- | --- | --- | --- | --- | --- | --- |
| Day | N | CR peak time (s),  mean (SD) | N | CR peak time (s),  mean (SD) | Estimated difference  [95%CI] | P-value | Adjusted  p-value |
| 1 | 1 | 380 (NA) | 5 | 249 (45) | -92 [-231, 46] | 0.19 | 1.00 |
| 2 | 9 | 250 (57) | 10 | 261 (17) | -5 [-35, 24] | 0.73 | 1.00 |
| 3 | 10 | 294 (45) | 17 | 278 (29) | -14 [-37, 9] | 0.22 | 1.00 |
| 4 | 14 | 281 (22) | 17 | 295 (37) | 8 [-11, 28] | 0.40 | 1.00 |
| 5 | 15 | 293 (31) | 18 | 286 (27) | -11 [-29, 7] | 0.21 | 1.00 |
| 6 | 19 | 316 (39) | 19 | 301 (26) | -11 [-30, 7] | 0.21 | 1.00 |
| 7 | 20 | 301 (30) | 20 | 300 (43) | -1 [-20, 17] | 0.89 | 1.00 |
| 8 | 21 | 304 (43) | 20 | 300 (34) | -4 [-24, 16] | 0.67 | 1.00 |
| 9 | 21 | 299 (47) | 20 | 289 (34) | -5 [-27, 17] | 0.63 | 1.00 |
| 10 | 21 | 301 (46) | 20 | 284 (32) | -14 [-38, 11] | 0.26 | 1.00 |

*† The degrees of freedom for all comparisons is 39. WT = wild type.*

**Supplementary Table 7. Proportions of “perfectly timed” CRs per training day and genotype for the 250 ISI training condition.**

|  | WT (n=21) | Nf1+/- (n=20) | Comparisons |  |  |
| --- | --- | --- | --- | --- | --- |
| Day | CR proportion,  median (q25-q75) | CR proportion,  median (q25-q75) | *Nf1^+/-^* / WT  OR [95%CI] | P-value | Adjusted  p-value |
| 1 | 0.00 (0.00-0.00) | 0.00 (0.00-0.01) | 22322438.25 [0-Inf] | 0.98 | 1.00 |
| 2 | 0.00 (0.00-0.10) | 0.04 (0.00-0.59) | 7.29 [1.55-34.22] | 0.01 | 0.12 |
| 3 | 0.00 (0.00-0.31) | 0.26 (0.09-0.62) | 5.13 [1.34-19.58] | 0.02 | 0.15 |
| 4 | 0.20 (0.00-0.47) | 0.35 (0.11-0.56) | 2.06 [0.64-6.59] | 0.22 | 0.92 |
| 5 | 0.44 (0.00-0.56) | 0.51 (0.43-0.72) | 2.74 [1.01-7.44] | 0.05 | 0.34 |
| 6 | 0.21 (0.07-0.47) | 0.40 (0.33-0.57) | 2.57 [1.08-6.08] | 0.03 | 0.26 |
| 7 | 0.35 (0.21-0.53) | 0.51 (0.30-0.64) | 1.68 [0.80-3.50] | 0.17 | 0.92 |
| 8 | 0.44 (0.24-0.69) | 0.53 (0.34-0.62) | 1.50 [0.78-2.88] | 0.23 | 0.92 |
| 9 | 0.55 (0.27-0.71) | 0.56 (0.37-0.71) | 1.20 [0.65-2.24] | 0.56 | 1.00 |
| 10 | 0.56 (0.40-0.72) | 0.67 (0.55-0.74) | 1.61 [0.84-3.08] | 0.15 | 0.92 |

*WT = wild type.*

#### Supplementary Table 8. Comparison of the percentage of clean sweep trials between genotypes per day.

|  | WT (n=24) | *Nf1^+/-^* (n=23) | Comparisons | | |
| --- | --- | --- | --- | --- | --- |
| Session | Clean sweeps (%),  median (q25-q75) | Clean sweeps (%),  median (q25-q75) | *Nf1^+/-^* / WT  OR [95%CI] | P-value | Adjusted  p-value |
| 1 | 1 (0-7) | 0 (0-5) | 0.84 [0.19, 3.75] | 0.82 | 1.00 |
| 2 | 7 (0-18) | 2 (0-7) | 0.41 [0.10, 1.66] | 0.21 | 1.00 |
| 3 | 4 (0-24) | 2 (0-14) | 0.57 [0.14, 2.22] | 0.41 | 1.00 |
| 4 | 10 (0-47) | 5 (0-27) | 0.57 [0.14, 2.26] | 0.42 | 1.00 |
| 5 | 14 (4-45) | 12 (2-38) | 0.82 [0.19, 3.45] | 0.79 | 1.00 |

*OR = odds ratio; WT = wild type.*

#### Supplementary Table 9. Percentage of correct steps, excluding clean sweep trials.

|  | WT (n=24) | *Nf1^+/-^* (n=23) | Comparisons | | |
| --- | --- | --- | --- | --- | --- |
| Session | Correct steps (%),  median (q25-q75) | Correct steps (%),  median (q25-q75) | *Nf1^+/-^* / WT  OR [95%CI] | P-value | Adjusted  p-value |
| 1 | 68 (59-75) | 60 (54-71) | 0.85 [0.61, 1.18] | 0.34 | 1.00 |
| 2 | 76 (68-82) | 73 (65-81) | 0.88 [0.63, 1.22] | 0.43 | 1.00 |
| 3 | 79 (70-87) | 75 (69-86) | 0.86 [0.61, 1.21] | 0.40 | 1.00 |
| 4 | 81 (71-86) | 79 (71-87) | 0.84 [0.58, 1.21] | 0.35 | 1.00 |
| 5 | 82 (78-88) | 83 (75-89) | 0.86 [0.57, 1.29] | 0.47 | 1.00 |

*OR = odds ratio; WT = wild type.*

#### Supplementary table 10. Latency to fall (s) on the Accelerated Rotarod per genotype, day and trial.

| Day | Block | Genotype | Subjects (n) | Latency (s),  Median (q25-q75) |  |
| --- | --- | --- | --- | --- | --- |
| 1 | 1 | *Nf1^+/-^* | 24 | 106 (83-123) | |
| 1 | 1 | WT | 23 | 112 (78-125) | |
| 1 | 2 | *Nf1^+/-^* | 24 | 136 (104-168) | |
| 1 | 2 | WT | 23 | 129 (95-185) | |
| 1 | 3 | *Nf1^+/-^* | 24 | 160 (131-182) | |
| 1 | 3 | WT | 23 | 184 (104-206) | |
| 1 | 4 | *Nf1^+/-^* | 24 | 184 (162-216) | |
| 1 | 4 | WT | 23 | 138 (131-195) | |
| 2 | 1 | *Nf1^+/-^* | 24 | 200 (143-215) | |
| 2 | 1 | WT | 23 | 152 (130-233) | |
| 2 | 2 | *Nf1^+/-^* | 24 | 187 (148-229) | |
| 2 | 2 | WT | 23 | 217 (165-255) | |
| 2 | 3 | *Nf1^+/-^* | 24 | 207 (161-256) | |
| 2 | 3 | WT | 23 | 208 (161-259) | |
| 2 | 4 | *Nf1^+/-^* | 24 | 207 (177-250) | |
| 2 | 4 | WT | 23 | 240 (170-278) | |
| 3 | 1 | *Nf1^+/-^* | 24 | 205 (157-260) | |
| 3 | 1 | WT | 23 | 214 (176-267) | |
| 3 | 2 | *Nf1^+/-^* | 24 | 224 (191-286) | |
| 3 | 2 | WT | 23 | 230 (200-277) | |
| 3 | 3 | *Nf1^+/-^* | 9 | 256 (252-290) | |
| 3 | 3 | WT | 9 | 276 (250-300) | |
| 3 | 4 | *Nf1^+/-^* | 9 | 214 (201-271) | |
| 3 | 4 | WT | 9 | 287 (223-300) | |
| 4 | 1 | *Nf1^+/-^* | 24 | 202 (169-257) | |
| 4 | 1 | WT | 23 | 213 (172-267) | |
| 4 | 2 | *Nf1^+/-^* | 24 | 221 (166-298) | |
| 4 | 2 | WT | 23 | 240 (190-300) | |
| 4 | 3 | *Nf1^+/-^* | 9 | 247 (207-300) | |
| 4 | 3 | WT | 9 | 300 (243-300) | |
| 4 | 4 | *Nf1^+/-^* | 9 | 258 (202-263) | |
| 4 | 4 | WT | 9 | 300 (241-300) | |

*WT = wild type.*

#### Supplementary Table 11. Latency to fall (s) on the Accelerated Rotarod per genotype and day.

|  | WT (n=23) | | *Nf1^+/-^* (n=24) | | Comparisons | | |
| --- | --- | --- | --- | --- | --- | --- | --- |
| Day | Observations  (n) | Latency (s),  median (q25-q75) | Observations (n) | Latency (s),  median (q25-q75) | *Nf1^+/-^* / WT  OR [95%CI] | P-value | Adjusted  p-value |
| 1 | 92 | 132 (96-187) | 96 | 144 (104-180) | 0.79 [0.33, 1.88] | 0.60 | 1.00 |
| 2 | 92 | 215 (143-263) | 96 | 200 (151-248) | 1.00 [0.42, 2.37] | 0.99 | 1.00 |
| 3 | 64^†^ | 245 (197-290) | 66^†^ | 224 (189-278) | 0.93 [0.38, 2.29] | 0.88 | 1.00 |
| 4 | 64^†^ | 241 (189-300) | 66^†^ | 224 (172-289) | 1.39 [0.56, 3.46] | 0.48 | 1.00 |

*An odds ratio (OR) of >1 indicates an increased risk of falling for the Nf1+/- mice over the wild type (WT) mice.* † *Day 3 and 4 contain less observations, as for those days session 3 and 4 were completed by only 9 out of 23 WT and 9 out of 24 Nf1+/- mice* ***(Supplementary Table 10)****.*

#### Supplementary Table 12. Time on beam (s) per beam width and genotype.

|  | WT (n=24) | *Nf1^+/-^* (n=23) | Comparisons^†^ | | |
| --- | --- | --- | --- | --- | --- |
| Beam width (mm) | Time on beam (s),  median (q25-q75) | Time on beam (s),  median (q25-q75) | Estimated difference (s),  [95%CI] | P-value | Adjusted  p-value |
| 6 | 10.44 (6.63-13.71) | 11.55 (9.21-18.61) | 2.98 [-0.03, 5.98] | 0.048 | 0.097^#^ |
| 12 | 7.65 (5.27-11.12) | 9.11 (7.46-11.27) | 1.65 [-0.57, 3.86] | 0.14 | 0.28 |

*The data contains two observations for each mouse per beam width. † The degrees of freedom for all comparisons is 45. ^#^ Trend towards a significant difference between genotypes of p<0.1. WT = wild type.*

**Supplementary**
